# Translational balancing questioned: Unaltered glycosylation during disulfiram treatment in mannosyl‐oligosaccharide alpha‐1,2‐mannnosidase‐congenital disorders of glycosylation (MAN1B1‐CDG)

**DOI:** 10.1002/jmd2.12213

**Published:** 2021-03-20

**Authors:** Lisa Kemme, Marianne Grüneberg, Janine Reunert, Stephan Rust, Julien Park, Cordula Westermann, Yoshinao Wada, Oliver Schwartz, Thorsten Marquardt

**Affiliations:** ^1^ University Children's Hospital Münster Muenster Germany; ^2^ Department of Clinical Sciences, Neurosciences Umeå University Umeå Sweden; ^3^ Gerhard‐Domagk‐Institute of Pathology University Hospital Muenster Muenster Germany; ^4^ Osaka Medical Center and Research Institute for Maternal and Child Health Osaka Japan

**Keywords:** MAN1B1‐CDG, disulfiram

## Abstract

MAN1B1‐CDG is a multisystem disorder caused by mutations in *MAN1B1*, encoding the endoplasmic reticulum mannosyl‐oligosaccharide alpha‐1,2‐mannnosidase. A defect leads to dysfunction within the degradation of misfolded glycoproteins. We present two additional patients with MAN1B1‐CDG and a resulting defect in endoplasmic reticulum‐associated protein degradation. One patient (P2) is carrying the previously undescribed p.E663K mutation. A therapeutic trial in patient 1 (P1) using disulfiram with the rationale to generate an attenuation of translation and thus a balanced, restored ER glycoprotein synthesis failed. No improvement of the transferrin glycosylation profile was seen.

## 1 INTRODUCTION

Congenital disorders of glycosylation (CDG) are an expanding group of inherited multisystem disorders affecting glycoprotein and glycolipid glycan synthesis and attachment. Over 125 different subtypes have been described so far, showing a diverse clinical spectrum.^1^, ^2^ Among these, disorders of *N*‐glycosylation represent the most common subgroup.^3^

**FIGURE 1:**
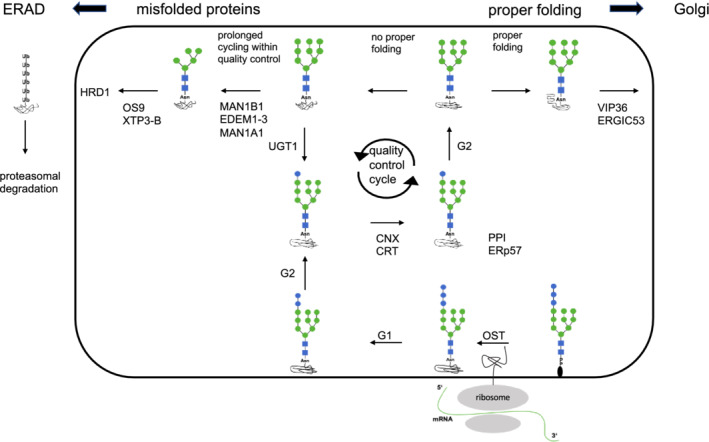
*N*‐glycan processing and endoplasmic reticulum‐associated protein degradation (ERAD). Blue square: *N*‐acetylglucosamine; green circle: mannose; blue circle: glucose. Preassembled lipid linked oligosaccharide is transferred to the nascent polypeptide chain entering the endoplasmatic reticulum, performed by oligosaccharyltransferase (OST).^53^, ^54^ Two glucose residues of the triglucosylated glycan are trimmed from branch A by ER alpha glucosidase 1 (G1) and subsequently ER alpha glucosidase 2 (G2). Lectin‐type chaperone clanexin (CNX) and its soluble paralogue calreticulin (CRT) recognize the Glc_1_Man_9_GlcNAc_2_ structure and support co‐ and posttranslational protein folding.^55^ ERp57 (oxidoreductase) and peptidyl‐prolyl isomerase cyclophilin B (PPI) are associated with CNX and CRT to promote proper folding by formating intramolecular and intermolecular disulphide bonds. Removal of the innermost glucose residue by G2 leads to a dissociation form the CNX/CRT chaperon system.^56^ Proteins which have folded and oligomerized properly are directed to the cis Golgi with the potential assistance of the mannose‐binding lectin ERGIC53, VIP36 and other homologous proteins.^57^ If proteins fail to aquire their correct conformation, folding sensor UGT1 recognizes the structure of the polypeptide with exposed non‐native determinants. If these are deteced, one glucose residue is added to the N‐glycane at branche A leading to a retruning entrance into the CNX/CRT cycle to achieve proper folding.^58^ Prolonged residence of misfolded glycoproteins within the CNX/CRT cycle promotes trimming of alpha 1,2‐linked mannose residues by MAN1B1 and EDEMS indicating failure of the glycoprotein to achieve native structure within a time frame.^5^, ^9^ N‐glycans with trimmed, lower mannoses, which expose an alpha 1,6 linked mannose on branche C are recognized by OS9 (mammal)/ Yos9p (yeast) and XTP3‐B.^59^, ^60^ These two ER resident ERAD lectins interact with the membrane‐embedded ubiquitin ligase HRD1 (HMG‐CoA reductase degradation protein 1) leading to a delivery of terminal misfolded proteins to dislocatin sites in the ER membrane.^61^, ^62^ This is folled by transport into the cytosol with polyubiquitination and proteasomal degradation.^63^

MAN1B1‐CDG is a multisystem disorder caused by mutations in *MAN1B1*, encoding the endoplasmic reticulum mannosyl‐oligosaccharide alpha‐1,2‐mannnosidase (MAN1B1). A defect leads to impaired degradation of misfolded glycoproteins.^4^, ^5^ Terminally misfolded or unassembled proteins are degraded by a process termed endoplasmic reticulum‐associated protein degradation (ERAD) (Figure 1: N‐glycan processing and ERAD). It serves as a part of the quality control in the early secretory pathway to prevent the accumulation of misfolded glycoproteins in the ER.^6^, ^7^ ERAD consists of three functional steps. MAN1B1 as a class *α*1,2‐mannosidase is involved in the first step, the recognition of a misfolded glycoprotein and a following extensive demannosylation by removing *α*1,2‐linked mannoses preferentially from branch B of the oligosaccharide to yield Man_8_GlcNAc_2._ ^8^, ^9^ Attributable to higher enzyme concentration or prolonged digestion, further mannoses can be trimmed.^5^, ^10^, ^11^

The second step is the retrotranslocation of the glycoprotein to the cytoplasm followed by an ubiquitin‐mediated degradation via the 26S‐proteasome.^12^

To date, there is no therapeutic approach to treat MAN1B1‐CDG. Influencing ER glycoprotein synthesis with the aim of “rationalizing” this process in the context of hindered glycosylation, also termed translational balancing, has been proposed as a possible approach to treat glycosylation disorders. Regarding this concept it was previously demonstrated that the acetaldehyde dehydrogenase inhibitor disulfiram, which was initially used for the treatment of alcohol dependence, is able to inhibit protein synthesis while promoting an extension of lipid linked oligosaccharides (LLO).^13^ Therefore, a therapeutic trial using disulfiram with the rationale to generate an attenuation of translation and thus a balanced, restored ER glycoprotein synthesis was conducted.

## 2 MATERIAL AND METHODS

### 2.1 Sample collection

Samples from both patients as well as healthy controls were collected after written informed consent was obtained and according to local bioethical regulations.

### 2.2 Genetic analysis

DNA was isolated from EDTA blood with the PAXGene Blood DNA System (PreAnalytiX GmbH, Hombrechtikon, Switzerland) and DNA concentration was measured with the Qubit 2.0 fluorometer (Thermo Fisher Scientific, Waltham, Massachusetts). Whole‐exome sequencing was performed with Illumina HiSeq2500/4000 as previously described.^14^ In silico analysis of the p.E663K substitution was performed with SIFT,^15^ PolyPhen‐2,^16^ MutationTaster,^17^ and HOPE.^18^

### 2.3 Glycosylation studies

Isoelectric focusing (IEF) was performed on a Pharmacia Phast system (Pharmacia Fine Chemicals, Uppsala, Sweden) according to a previously published protocol.^19^ SDS‐PAGE of serum transferrin following immunoprecipitation was performed following the previously reported method.^20^

High‐performance liquid chromatography (HPLC) of carbohydrate‐deficient transferrin was performed using the commercially available “CDT in serum” kit (Chromsystems Instrument and Chemicals GmbH, Gräfelfing, Germany) according to the manufacture's protocol using capillary blood samples collected with Microvette CB 300 LH, 100 μL (SARSTEDT AG & Co. Nümbrecht, Germany), a system for capillary blood collection as described previously.^21^

### 2.4 Matrix‐assisted laser desorption time‐of‐flight mass spectrometry of transferrin

Mass spectrometry (MS) of glycopeptides for glycoform profiling was performed according to the method described before with some modifications.^22^ Transferrin was purified from serum by immunoaffinity with rabbit polyclonal antibody against human transferrin. Purified transferrin was dissolved in 0.5 mL of 6 M guanidium hydrochloride, 0.25 M Tris‐HCl, pH 8.5 and reduced with 5 mg of dithiothreitol at 60°C for 3 hours. Then, 9 mg of iodoacetamide were added to the solution, followed by incubation in the dark at room temperature for 30 minutes for carbamidomethylation. The reagents were removed by a gel fitration column, NAP‐5 (GE Healthcare, Piscataway, New Jersey), equilibrated with 0.05 N HCl, and the recovered protein solution was adjusted at pH 8.5 with Tris. The carbamidomethylated transferrin was digested by a mixture of trypsin and *Acromobacter* lysylendopeptidase (Wako, Osaka, Japan) at 37°C for 12 hours. The procedure of glycopeptide enrichment was omitted. The digest was desalted using a Millipore ZipTip C18 pipette tip and analyzed with a matrix‐assisted laser desorption time‐of‐flight (MALDI TOF) mass spectrometer (Voyager DE‐Pro). The sample matrix was 20 mg/mL of 2,5‐dihydroxybenzoic acid dissolved in 50% acetonitrile in water. Measurements were performed in positive ion and linear TOF mode.

### 2.5 Electron microscopy

Dermal fibroblasts were collected from patient 1 (P1) using 4 mm punch biopsy and cultivated under standard conditions. Fibroblast pellets were fixed in 2,5% glutaraldehyde with Sörensen phosphate buffer. Subsequently, the samples were fixed with 1% osmiumtetroxide, dehydrated and embedded in Epon. Then, 60 nm ultrathin sections were cut by Leica Ultracut R ultramicrotome (Vienna, Austria) and counterstained with 8% uranyl acetate in bidistilled water and incubated with lead citrate solution. Samples were inspected with a CM 10 transmission electron microscope (Philips, Amsterdam, Netherlands). The evaluation of the dilatation of the Golgi apparatus was performed optically. Then, 72 Golgi complexes of P1 and 62 Golgi complexes of the control were analyzed.

### 2.6 Disulfiram therapy

Patient 1 received disulfiram (L. Molteni & C. dei F.lli Alitti Societa di Esercizio, Scandicci, Italy). Mannitol‐Siliciumdioxid NRF was used as filling agent with 10 mg/20 mg of disulfiram powder. The capsules were manufactured by Bookholter Apotheke OHG (Nordhorn, Germany).

The FDA‐recommended dose range for disulfiram with regard to adults is 125 mg/d to a maximum of 500 mg/d (average daily dose of 250 mg).^23^

The target dose was weight‐adapted to the adult dose (weight of P1: 35 kg, defined maximum dose: 150 mg/d).

Disulfiram was administered orally in increasing doses from 10 to 150 mg once a day with an increment approximately every 4 weeks. We increased the dosage in the setting of an extended up‐titration in a series of seven steps, initially by 10 mg in order to quickly detect possible side effects. Monitoring was conducted in the context of clinical presentations. Adverse events including fatigue, headache, hepatic damage, allergic dermatitis, peripheral neuropathy, and mental status changes are rarely reported.^23^ A meta‐analysis of 22 studies regarding to safety and tolerance of disulfiram shows no difference between disulfiram and control groups reporting serious adverse events requiring hospitalization or lead to death.^24^

With good clinical tolerability we raised in the further progression by 30 mg and at last 50 mg. Glycosylation was assessed eight times using HPLC.

## 3 RESULTS

### 3.1 Case report

**FIGURE 2:**
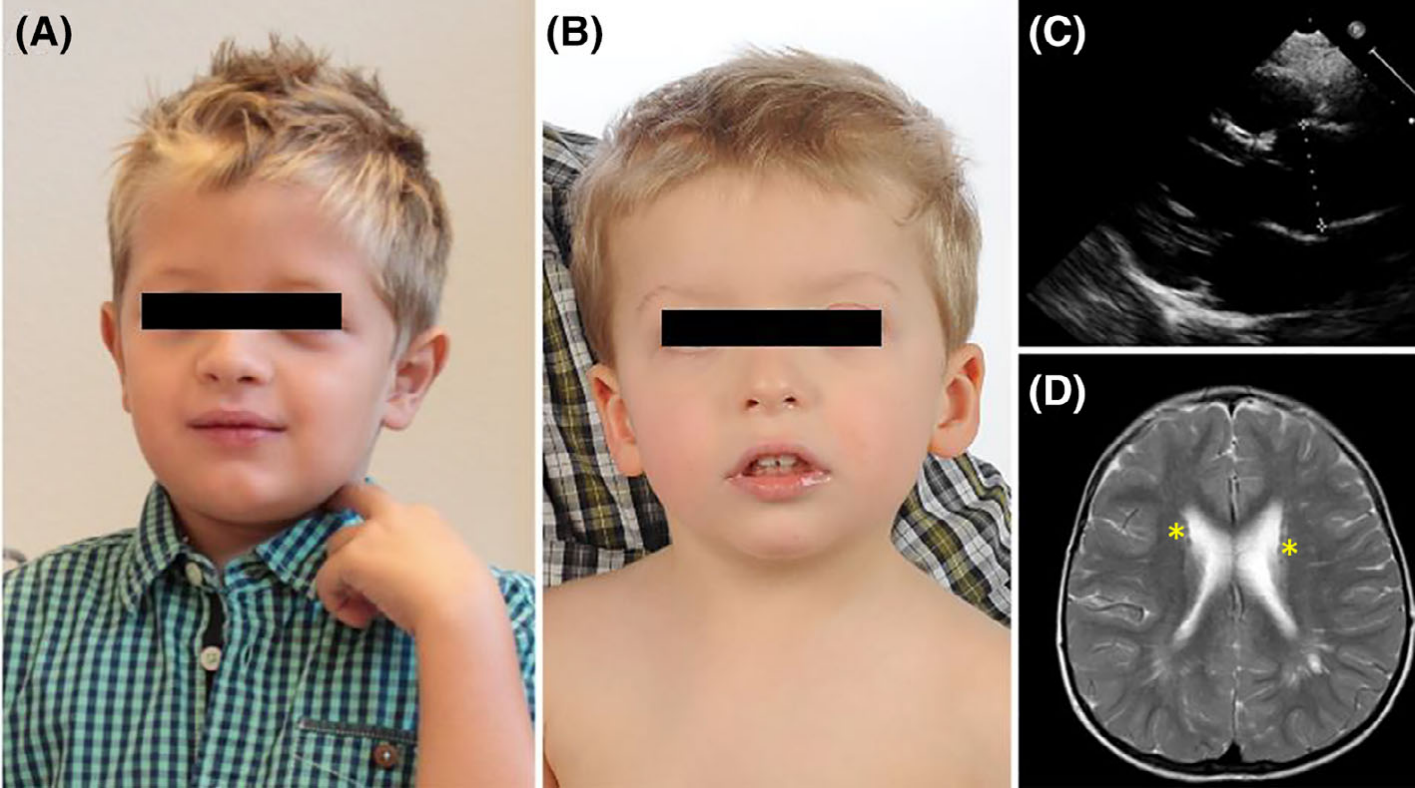
Clinical presentation of mannosyl‐oligosaccharide alpha‐1,2‐mannnosidase (MAN1B1)‐congenital disorders of glycosylation (CDG). A, Picture of patient 1 (P1). B, Picture of patient 2 (P2). C, Echocardiography of P1 in the parasternal longitudinal axis shows a slight extension of the aortic bulbous (28 mm; 28 mm/m^2^ body surface area; reference value 15‐20 mm/m^2^ body surface area). D, Neurological findings of subject P 2: A magnetic resonance image (MRI) in T2. The image revealed multiple subependymal heterotopia of the gray matter, exemplarily marked with yellow stars

Patient 1 (P1) is a 10‐year‐old son of non‐consanguineous parents of German origin, diagnosed at the age of 8 years. Muscular hypotonia was present directly after birth, leading to delayed motor development (sitting at 13 months, unassisted walking at 2 years and 11 months). Delayed speech development in combination with progressive dysarthria was noted. Furthermore, the patient was diagnosed with annuloaortic ectasia, which has been reported in few other cases^25^ (Figure 2: clinical presentation of MAN1B1‐CDG).

Typical dysmorphic features of the disorder like down‐slanting palpebral fissures and low‐set eats were present, albeit less pronounced than in other described cases. In addition, a right‐hand simian crease and plano‐valgus feet were noted. Brain MRI showed no structural abnormalities while EEG revealed nonspecific mild global changes without any signs of convulsive activity. GOT was mildly elevated ranging from 88 to 147 mg/dL (reference: 10‐50 mg/dL).

Patient 2 (P2) is the 5‐year‐old son of an unrelated couple of German origin, diagnosed at the age of 5 years. As in patient 1, muscular hypotonia was present directly after birth. Delayed motor and speech development were noted (unassisted walking at 3 years and 6 months, one‐word sentences at 2 years and 6 months). He presented with characteristic facial dysmorphism: hypertelorism, sparse eyebrows, down‐slanting palpebral fissures, an epicanthus and large lower set ears. In addition, clinodactyly of the fifth finger on both sides was noted. At the age of 1 year, a stroke‐like episode with left‐sided hemiparesis occurred. Brain‐MRI showed no signs of ischemia or intracranial hemorrhage, while subependymal gray matter heterotopia and prominent Virchow‐Robin spaces were present (Figure 2: clinical presentation of MAN1B1‐CDG). Heterotopia was described in one additional case of MAN1B1‐CDG.^23^, ^26^ EEG showed no seizure activity or post‐convulsive changes during the episode. Control EEG showed increased susceptibility to seizures with sharp‐waves and slow waves with a theta activity of 6 to 7 Hz. As in P1, slight elevation of GOT was present (74‐94 mg/dL).

### 3.2 Genetic analysis

**FIGURE 3:**
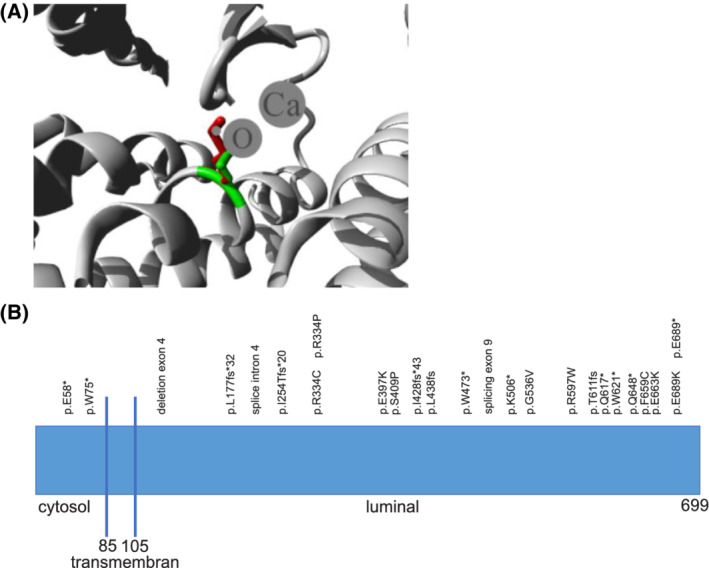
Protein mannosyl‐oligosaccharide alpha‐1,2‐mannnosidase (MAN1B1) and known mutations. A, Close‐up of the mutation p.E663K. The protein is colored gray, the side chains of both the wild type and the mutant residue are shown and colored green and red, respectively (https://www3.cmbi.umcn.nl/hope/report/5e787bb79cd87612add919e0/). The affected residue is involved in Ca^2+^ binding via hydrogen bonds.^8^ B, Protein MAN1B1 and known mutations. *MAN1B1* encoding the mannosyl oligosaccharide *α*1,2‐mannosidase is localized on chromosome 9q34.3 and consists of 13 coding exones.^5^ The encoded protein consists of an N‐terminal cytoplasmic tail (85 amino acids), a transmembrane helix (17 amino acids), a luminal stem domain (137 amino acids) and a luminal catalytic domain (341 amino acids)^5^, ^9^

Whole‐exome sequencing identified compound heterozygous mutations within *MAN1B1* in each case. Patient 1 was found to be compound heterozygous *in trans* for the previously described *MAN1B1* variants c.1189G > A (p.E397K) and c.2065G > A (p.E689K) with the former leading to no measurable enzyme activity in a homozygous state explainable by a reduced expression or stability of this protein variant while the latter affects the active site of the protein.^27^, ^28^ In patient 2, the previously described variant c.1789C > T (p.R597W)^28^ was identified in trans with the undescribed variant c.1987G > A (p.E663K). This variant lies in a highly conserved region and is predicted to be pathogenetic by all used prediction algorithms.^18^ The affected residue is involved in Ca^2+^ binding via hydrogen bonds^8^ (Figure 3: protein MAN1B1 and known mutations).

### 3.3 Glycosylation assays

To investigate the glycosylation profile of the patients, analysis of serum transferrin by HPLC, IEF and sodium dodecyl sulfate polyacrylamide gel electrophoresis (SDS‐PAGE) were performed. Glycan structure was analyzed using MALDI TOF MS.

**FIGURE 4:**
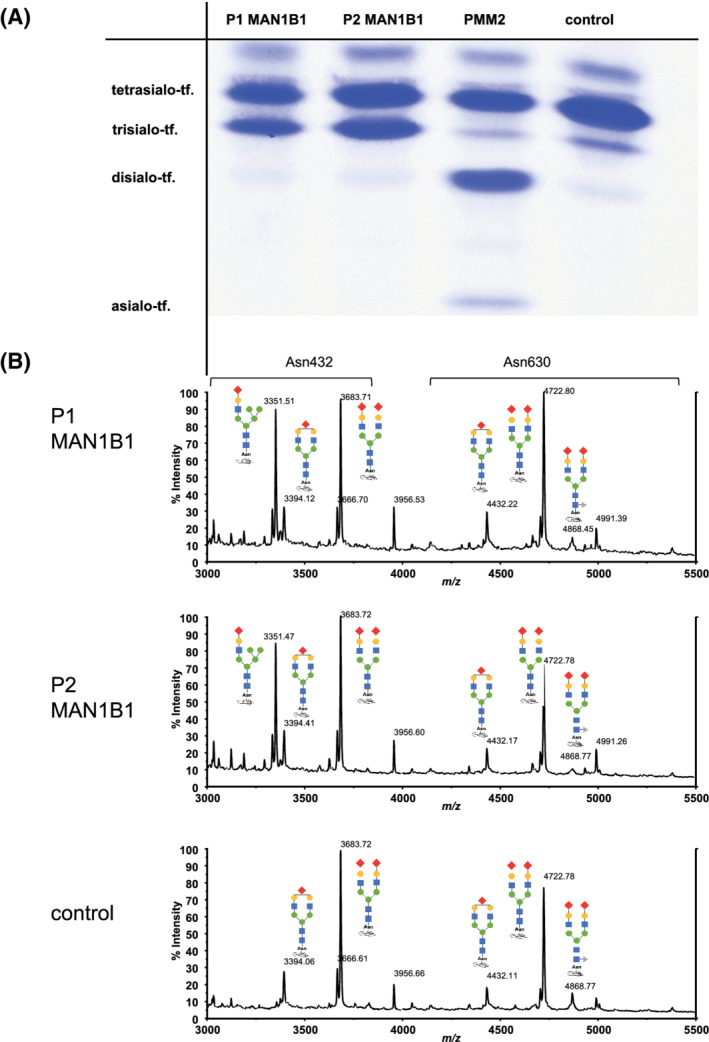
Glycosylation assays. A, Isoelectric focusing (IEF) showed a type 2 pattern with an increased trisialo‐transferrin. A PMM2‐congenital disorders of glycosylation (CDG) patient showed an increased disialo‐transferrin and asialo‐transferrin fraction. B, Matrix‐assisted laser desorption time‐of‐flight mass spectrometry (MALDI TOF MS) of P1 and P2. A Hybrid‐type glycan is found only at Asn432 site of transferrin in mannosyl‐oligosaccharide alpha‐1,2‐mannnosidase (MAN1B1). This major species with *m/z* 3351 is not found in the control. It is corresponding to the glycan type Sia_1_Gal_1_Man_5_GlcNAc_3_. Blue square: *n*‐acetylglucosamine; green circle: mannose; blue circle: glucose; yellow circle: galactose; red rhombus: sialic acid; gray triangle: fucose

Both subjects show a type 2 serum transferrin IEF pattern with an increase of trisialo‐transferrin (Figure 4: glycosylation assays).

HPLC revealed an increased trisialo‐transferrin (P1: 45,5%; P2:42,3%; reference <6,5%), instead tetrasialo‐transferrin (P1: 51,9%; P2: 54,8%; reference >85%) and pentasialo‐transferrin (P1: 2,5%, P2: 2,7%; reference >15%) were decreased (supplement: HPLC).

Immunoprecipitation and SDS‐PAGE of serum transferrin showed no observable difference when compared to wild‐type controls (supplement: Immunoprecipitation and SDS‐PAGE).

MALDI TOF MS of serum transferrin detected a hybrid‐type glycan structure only at Asn432 site of transferrin in MAN1B1, as shown before^22^ (Figure 4: glycosylation assays).

### 3.4 Electron microscopy

**FIGURE 5:**
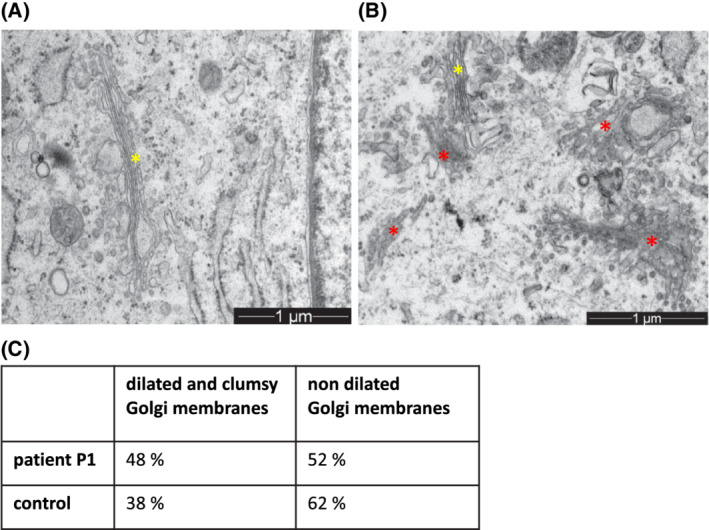
Electron microscopy. A,B, Electron microscopy of fibroblasts of the control (picture A) and patient 1 (picture B). Yellow stars show regular Golgi morphology with flattened stacked membranes. Red stars represent an altered Golgi morphology with dilated cisterns and a widened Golgi appearance. C, optical analysis of electron microscopy

We performed electron microscopy of the fibroblasts of patient 1 (P1) to evaluate the Golgi apparatus optically. The fibroblasts of the patient reveal a more heterogeneous pattern of Golgi cisterns compared to the control. The individual membrane‐bound cisterns appear in half of the examined Golgi complex clumsy and dilated (Figure 5: electron microscopy).

Some dilated dictyosomes also occur in the control, but less frequently than in the patient.

### 3.5 Disulfiram therapy

**FIGURE 6:**
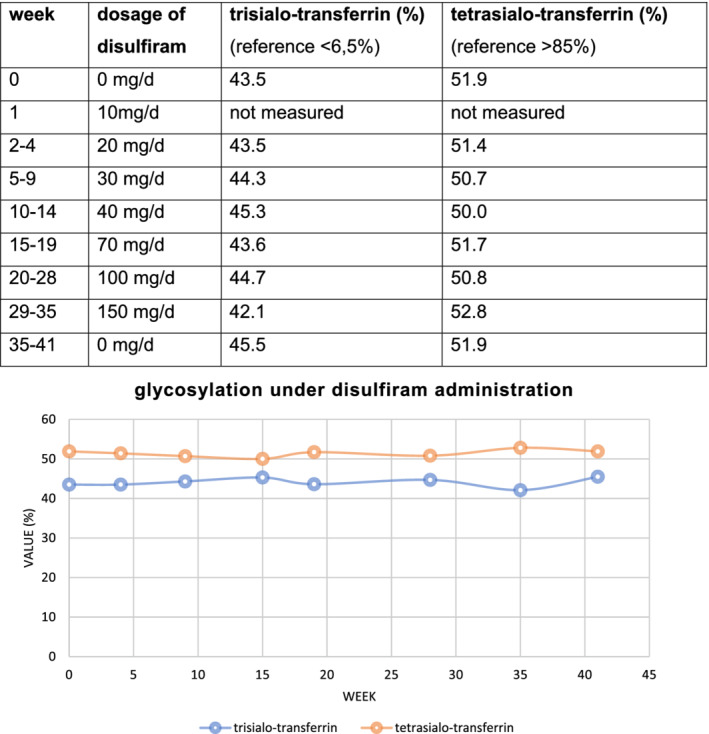
Disulfiram administration. Disulfiram was administered orally in increasing doses from 10 to 150 mg/d once a day with an increment approximately every 4 weeks. Glycosylation was assessed using high‐performance liquid chromatography (HPLC)

Within our therapy trial, no normalization of the transferrin glycosylation profile was detected at any of the administered dosages. The corresponding HPLCs of P1 remained constant with minor variations (Figure 6: disulfiram administration).

## 4 DISCUSSION

MAN1B1‐CDG is a growing subgroup within the family of CDG. We report on two new patients, who presented with a general developmental delay and characteristic facial dysmorphism. Whole‐exome sequencing revealed the previously undescribed variant c.1987G > A; [p.E663K] in addition to known mutations. Both patients were compound heterozygous.

Since the first description of MAN1B1‐CDG by Rafiq et al, an increasing number of cohorts have been reported.^27^ The clinical presentation of MAN1B1‐CDG is mainly characterized by a typical face, mostly moderate intellectual disability, behavioral disturbances and obesity.^25^, ^26^, ^27^, ^28^, ^29^, ^30^, ^31^, ^32^, ^33^

Neurological manifestations are frequent with an abnormal MRI‐brain image, seizures and ataxia reported in several cases. Stroke like episodes, which occurred in one of our patients, have not been documented in MAN1B1‐CDG, but in other CDG diseases, for example, PMM2‐CDG. Recently, evidence has been presented that stroke like episodes in PMM2 CDG may occur due to hypoglycosylation of calcium channels.^34^ Also, ataxia might be explained by a hypoglycosylation driven channelopathy.^34^

Table 1 summarizes additional features sorted by genotype.

**TABLE 1:** Clinical features sorted by genotype

Genotype complementary DNA/protein	Number of patients/families	Intellectual disability	Neurological involvement	Skeletal and joint involvement	Organic abnormalities	Behavioral concern	Cohort
		Mild (50‐65 IQ)					
		Moderate (35‐49 IQ)					
		Severe (20‐34 IQ)					
c.172 G > T/p.E58* c.1225 T > C/p.S409P	1/1	1 moderate	NA	NA	NA	1 auto‐aggressivity 1 repetitive movement	Rymen et al^25^
c.224G > A/p.W75*	1/1	1 severe	1 ataxia	NA	NA	1 autism/anxiety/tic	van Scherpenzeel et al^28^
c.465 + 1460_620 + 527del/deletion of exon 4 c.1445 + 2delTGAG/splicing of exon 9	1/1	1 mild	1 multiple small white lesions	1 joint hypermobility 1 skin laxity	1 ventricle septum defect (spontaneous closure)	1 autism spectrum disorder	Rymen et al^25^
c.530_542del/p.L177fs*32 c.621‐2A > G/splice intron 4	2/1	1 mild 1 severe	2 seizure	1 joint hypermobility 1 skin laxity	NA	1 autism/anxiety/tics 1 aggressivity	van Scherpenzeel et al^28^
c.761_764del/p.I254Tfs*20 c.1000C > T/p.R334C	2/1	2 moderate	NA	NA	NA	1 aggressivity	Balasubramanian et al^26^
c.1000C > T/p.R334C	12/6	7 mild 3 mild—moderate 2 moderate	1 seizure 1 slight widening of outer ventricles 1 delayed myelinization 1 hearing lost unilateral	3 joint hypermobility 3 skin laxity 2 scoliosis 1 pectus excavatum 1 11 rib pairs	1 episode of hyperglycemia 1 reflux esophagitis 1 inguinal hernia 1 anal fissure	1 autism/anxiety/tics 2 aggressivity	Rafiq et al^27^ Rymen et al^25^ van Scherpenzeel et al^28^ Hoffjan et al^29^
c.1001G > C/p.R334P c.1849C > T/p.Q617*	1/1	1 moderate	NA	NA	GOT/GPT E PTT E AT3 D	1 aggressivity 1 autism/anxiety/tics	van Scherpenzeel et al^28^
c.1189 G > A/p.E397K	6/3	1 mild 4 mild—moderate 1 NK	1 seizure 1 white‐matter abnormality (cerebellum and cerebrum), prominent perivascular space in right parietal lobe	NA	NA	1 inappropriate sexualized behavior	Rafiq et al^27^
c.1189G > A/p.E397K c.2065G > A/p.E689K	1/1	1 moderate	NA	NA	1 ectasia of aortic bulb 1 GOT E	1 autism spectrum disorder	This study
c.1225 T > C/p.S409P	1/1	1 severe	1 cerebellar hypoplasia with vermian atrophy	NA	NA	NA	Rymen et al^25^
c.1225 T > C/p.S409P c.1282delA/p.I428fs*43	1/1	1 mild	NA	NA	1 GOT/GPT E	NA	van Scherpenzeel et al^28^
c.1311del/p.L438fs	2/1	2 moderate	1 periventricular heterotopia with overlying cortical dysplasia	1 scoliosis 1 toe syndactyly	NA	1 aggressivity	Balasubramanian et al^26^
c.1418 G > A/p.W473*	3/1	3 mild	1 seizure	1 clinodactyly of the fifth finger 1 toe syndactyly	NA	1 aggressivity	Rafiq et al^27^
c.1516A > T/p.K506*	2/1	NK	NA	NA	NA	NA	Kvarnung et al^32^
c.1607G > T/p.G536V	1/1	NK	NA	NA	NA	NA	Barbosa et al^33^
c.1789C > T/p.R597W c.2065G > A/p.E689K	2/1	1 moderate 1 NK	NA	NA	NA	NA	van Scherpenzeel et al^28^
c.1789C > T/p.R597W c.1987G > A/p.E663K	1/1	1 mild	1 subependymal heterotopia, prominent Virchow Robin spaces 1 stroke like episode	1 clinodactyly of the fifth finger	GOT E	NA	This study
c.1833_1834delAG/p.T611fs	1/1	1 mild	1 seizure	1 joint hypermobility 1 skin laxity 1 pectus excavatum	1 dilatation of aortic root	NA	Rymen et al^25^
c.1863G > A/p.W621*	2/1	2 NK	NA	NA	NA	1 aggressivity	van Scherpenzeel et al^28^
c.1942 C > T/p.Q648*	1/1	1 mild	NA	1 joint hypermobility 1 skin laxity 1 toe syndactyly 1 clinodactyly of the fifth finger	NA	NA	Bastaki et al^31^
c.1976 T > G/p.F659C	1/1	1 mild	1 ataxia	1 joint laxity 1 contractures	1 AT3 D 1 PTT E	1 autism/anxiety/tics	van Scherpenzeel et al^28^
c.2065G > T/p.E689*	1/1	1 moderate	1 mildly delayed myelinization	1 joint laxity	NA	NA	van Scherpenzeel et al^28^
c.1789C > T/p.R597W c.2065G > A/p.E689K	2/1	1 moderate 1 NK	NA	NA	NA	NA	van Scherpenzeel et al^28^

Abbreviations: AT3, antithrombin 3; D, decreased; E, elevated; GOT, glutamate oxaloacetate transaminase; GPT, glutamate pyruvate transaminase; NA, not applicable; NK, not know; PTT, partial thromboplastin time.

Comparing the already known mutations sites (Figure 3: protein MAN1B1 and known mutations), which are distributed evenly over the entire gene, there is no indication of mutation hotspots or a direct genotype‐phenotype correlation in the available data.

In general, previously described missense mutations reduce enzyme activity due to a decreased protein concentration. This leads to a delayed trimming from Man_9_GlcNAc_2_ to Man_8_GlcNAc_2_, with a minimal reported trimming efficiency of 36% of normal values.^25^, ^27^

The intracellular localization of MAN1B1 has been subject to debate. It was initially predicted to act as an ER‐resident protein while other studies indicate a localization of MAN1B1 within the Golgi apparatus of mammalian cells.^9^ The proposed model describes MAN1B1 as a checkpoint within the Golgi to recycle misfolded proteins that escaped ERAD.^35^, ^36^ In contrast, an alternative model suggests that MAN1B1 is located in specialized quality control vesicles (QVC) within the ER‐derived quality control compartment (ERQC). In ERQC is a higher concentration of MAN1B1 present, which is required for trimming to Man_5‐6_GlcNAc_2_ in vivo, leading to ERAD.^37^, ^38^, ^39^

The use of immunofluorescence methods leads to an artificial appearance of MAN1B1 in a Golgi pattern caused by membrane disturbance.

The described QVCs show a vesicular pattern and are highly mobile depending on microtubules and COP‐II, demonstrated by inhibitors of these, which significantly affect the mobility of QCVs.^38^ During ER stress, QCV assemble in a juxtanuclear region at the ERQC.^37^ It provides a high local enzyme concentration and accumulates ERAD substrates, which underlines the role of MAN1B1 in targeting misfolded substrates to ERAD.^40^

In the performed electron microscopy of P1's cells, the Golgi apparatus cisterns appear dilated and coarse. Golgi membranes are compressed, the single dictyosomes occur often only twice connected in series. A pre‐Golgi located defect at the ERQC might cause alterations of Golgi morphology, since a mutation of MAN1B1 results in an inadequate elimination of misfolded proteins. This toxic misfolded protein can lead to malfunctions in the following compartments and a disorder of cell homeostasis including an alteration of morphology.

### 4.1 Treatment of MAN1B1‐CDG

Despite recent advances, the vast majority of CDG lacks specific therapeutic approaches. In some, monosaccharide or cofactor supplementation have been shown to exert favorable effects on both glycosylation and clinical presentation.^20^, ^41^, ^42^, ^43^, ^44^, ^45^, ^46^, ^47^

To date, there is no therapeutic approach to treat MAN1B1‐CDG. Due to MAN1B1's mannose cleavage function, it is not possible to support enzyme function by creating an excess of substrate or removal of the resulting product to positively influence kinetics, as it is done in other CDG. The substitution of the co‐factor Ca^2+^ does not appear to be feasible due to the strict regulation of Ca^2+^ metabolism.

**FIGURE 7:**
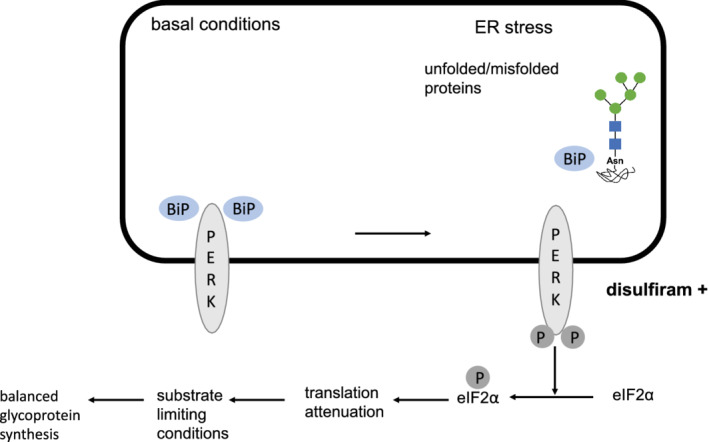
PKR‐like ER kinase (PERK) in the frame of unfolded protein response (UPR). Due to ER Stress, translational initiation is attenuated by phosphorylation of the eukaryotic initiation factor 2α (eIF2α).^64^, ^65^ The phosphorylation of eIF2α is mediated by PERK (PKR‐like endoplasmic reticulum kinase), which phosphorylates eIF2α at Ser 51 leading to a reduction of the polypeptide synthesis (70‐90%) and dimishing the load of ER client proteins. Under basal conditions, without stress, heat shock protein 90 and BiP bind to the cytoplasmic and ER luminal domains of PERK, leading to a stabilization and preventing its activiation. Under stress conditions BiP binds to unfolded and misfolded proteins, thus activating PERK by permitting the release of PERK for homodimeriziation and autophosphorylation. ^48^

Aside from substrate or cofactor substitution, influencing the ER glycoprotein synthesis with the aim of “rationalizing” this process in the context of hindered glycosylation has been proposed as a possible approach to treat glycosylation disorders.^13^ This concept, also dubbed translational balancing, aims to ameliorate ER glycoprotein synthesis by the activation of unfolded protein response (UPR) initiated by the PKR‐like ER kinase (PERK).^48^ Impaired *N*‐glycosylation causes an accumulation of LLO intermediates. Translational attenuation by PERK balances ER glycoprotein synthesis with LLO flux.^13^ It was previously demonstrated that the acetaldehyde dehydrogenase inhibitor disulfiram is able to stimulate PERK leading to an inhibition of protein synthesis while promoting LLO extension^13^ (Figure 7: PERK in the frame of UPR).

In our trial of oral disulfiram (DSF) therapy, no effect on transferrin glycosylation could be observed. The complex pharmacokinetic can be considered as a possible explanation.^23^, ^49^, ^50^ Disulfiram (C_10_H_20_N_2_S_4_) is rapidly metabolized and extremely unstable in gastric fluid and blood. After absorption, DSF is quickly reduced to its monomer diethyldithiocarbamic acid (DDC), followed by further conversion. The reduction to its metabolite DDC takes 4 minutes in blood in vitro.^51^ The drug has a strong affinity to bind albumin (96.1%) and a high lipid solubility. The plasma concentration after oral administration of a therapeutic dosage for adults (500 mg) is below the limit of detection. In vivo, the main peak plasma concentration reaches an nM concentration after 9.2 hours (DSF), respectively.^52^

Disulfiram may only reach insufficient concentration in cells when administered orally or the generated metabolites are not able to influence glycoprotein synthesis positively. Regardless, it is conceivable that disulfiram is in general not sufficient to generate a proper glycosylation in MAN1B1‐CDG.

### 4.2 Outlook

With 46 patients, MAN1B1‐CDG belongs to the more frequent *N*‐glycosylation disorders, which forms an extra stimulus to search for a therapy.

## CONFLICT OF INTEREST

The authors declare no conflict of interests.

## INFORMED CONSENT

All procedures followed were in accordance with the ethical standards of the responsible committee on human experimentation (institutional and national) and with the Helsinki Declaration of 1975, as revised in 2000. Informed consent was obtained from all patients for being included in the study.

## Supporting information


**Appendix** **S1**: Supporting information
