# Supplementary material for: Translational balancing questioned: Unaltered glycosylation during disulfiram treatment in mannosyl‐oligosaccharide alpha‐1,2‐mannnosidase‐congenital disorders of glycosylation (MAN1B1‐CDG)
Source: JIMD Rep. 2021 Mar 20;60(1):42–55. doi: 10.1002/jmd2.12213 (PMC8260486; doi:10.1002/jmd2.12213)

**Supplement**

**HPLC**

HPLC of P1 and P2 shows an increase of trisialo-transferrin and a decrease of tetrasialo-transferrin and pentasialo-transferrin. Asialo-transferrin, monosialo-transferrin and disialo-transferrin were in the normal range.

**Immunoprecipitation and SDS-PAGE**

Immunoprecipitation and SDS-PAGE of serum Transferrin showed 2 carbohydrate chains, in accordance with the IEF values. A PMM2-CDG patient showed 0 and 1 carbohydrate chains in addition to the findings in the patient and control.


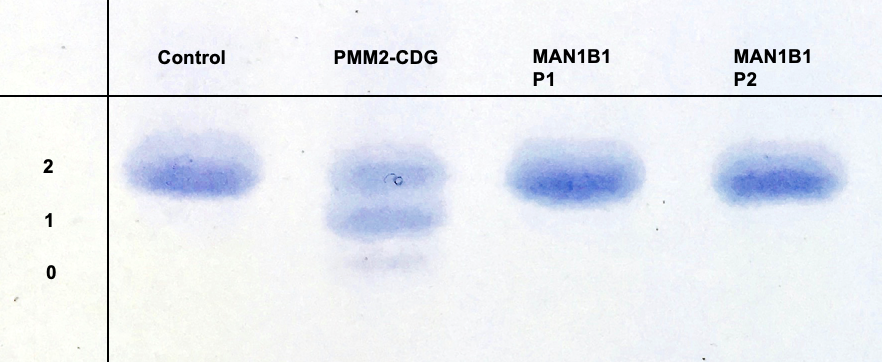

Supplement: Supplementary file 1 — Appendix S1: Supporting information [file JMD2-60-42-s001.docx]
